# Confidence Without Wisdom: The Dunning–Kruger Problem in Modern Surgery

**DOI:** 10.7759/cureus.102174

**Published:** 2026-01-23

**Authors:** Dimitrios Moris, Emmanouil Giorgakis

**Affiliations:** 1 Surgery, Medstar Georgetown Transplant Institute, MedStar Georgetown University Hospital, Washington, D.C., USA; 2 School of Medicine, European University Cyprus, Nicosia, CYP; 3 Surgery, University of North Carolina at Chapel Hill, Chapel Hill, USA

**Keywords:** cognitive bias, confidence level, dunning-kruger effect, self-assessment, surgical judgment

## Abstract

Confidence is indispensable in surgery, yet when it becomes misaligned with judgment it may place patients and professionals at risk. This editorial examines how confidence evolves across different stages of a surgical career and how miscalibration can emerge during periods of transition and increasing responsibility. Drawing on philosophical perspectives on knowledge and uncertainty alongside practical considerations of training and clinical practice, we explore how confidence may outpace reflective self-awareness and how experience gradually reshapes surgical judgment. We argue that miscalibrated confidence is not primarily an individual shortcoming but a predictable consequence of professional cultures and systems that emphasize decisiveness, productivity, and visible achievement. Understanding how confidence is acquired, reinforced, and recalibrated is essential for promoting patient safety, professional development, and ethically grounded surgical decision-making.

## Editorial

Introduction

Few professions depend on confidence as explicitly as surgery [1]. Surgeons are expected to decide, act, and assume responsibility in environments characterized by uncertainty, time pressure, and irreversible consequences. Yet confidence in surgery is not merely a personal trait; it is a professional currency, actively cultivated, rewarded, and often mistaken for competence. Within this culture, the Dunning-Kruger effect, the tendency of individuals with limited expertise to overestimate their abilities, while those with greater expertise increasingly recognize their limitations, assumes exceptional relevance [2]. Unlike many cognitive biases discussed in medicine, the Dunning-Kruger effect in surgery is neither subtle nor benign. It intersects directly with patient safety, training paradigms, and professional identity. Its consequences are amplified by hierarchical structures, delayed feedback loops, and incentive systems that favor decisiveness and productivity over reflection and restraint. This editorial explores the Dunning-Kruger effect in surgery from two complementary perspectives: that of an early-career surgeon navigating the transition to independent judgment, and that of a mid-career surgeon who has achieved technical success, professional recognition, and increasing leadership responsibility, while confronting the growing complexity of judgment that accompanies experience.

Surgical confidence as a cultural construct

Surgical confidence is often portrayed as an intrinsic attribute, something one either possesses or lacks. In reality, it is a socially constructed behavior, learned and reinforced through training. From the earliest stages of residency, surgeons are conditioned to project certainty. Presentations are expected to be definitive, operative plans decisive, and hesitation minimized. This culture serves an important function: indecision in the operating room can be dangerous. However, when confidence becomes performative rather than reflective, it ceases to track true competence. The Dunning-Kruger effect thrives in precisely such environments. Early technical success, often achieved under close supervision and favorable conditions, can foster an inflated sense of mastery [3]. Because negative feedback is frequently softened, delayed, or redirected toward system factors, individuals may lack the corrective signals necessary to recalibrate self-assessment. In surgery, where outcomes are probabilistic and complications multifactorial, overconfidence can persist for years before it is meaningfully challenged.

The early-career surgeon: when autonomy outpaces judgment

The transition from trainee to independent surgeon represents one of the most vulnerable phases for the manifestation of the Dunning-Kruger effect. Technical milestones have been met, credentials obtained, and external validation accumulated. Yet judgment, the ability to integrate patient factors, disease biology, technical risk, and ethical restraint, matures far more slowly than operative skill. For the early-career surgeon, confidence often functions as armor. In competitive academic and clinical environments, admitting uncertainty may be perceived as weakness. The result is defensive certainty: firm operative plans, narrow differentials, and resistance to dissent [4]. This posture is rarely malicious; it is adaptive within a system that rewards decisiveness and penalizes hesitation. Nevertheless, it is precisely in this phase that procedural fluency is most likely to be conflated with comprehensive competence. Philosophically, this reflects a failure of epistemic calibration [5]. Knowing how to operate is mistaken for knowing when not to. The Dunning-Kruger effect, in this context, is less about ignorance than about misaligned self-perception, an error reinforced by structural incentives rather than individual character.

The mid-career surgeon: recalibrating confidence through experience

At a later, but still highly active, stage of the professional spectrum, the mid-career surgeon often begins to embody a partial inversion of the Dunning-Kruger effect. By this point, technical confidence is well established, yet it is increasingly tempered by lived experience. With experience comes an accumulation of adverse outcomes, near-misses, and complications that never appear in databases or publications. Success becomes contextual rather than assumed, and confidence evolves into prudence. This form of confidence is frequently misunderstood. What appears as hesitation to trainees may reflect a sophisticated risk calculus informed by decades of pattern recognition. Mid-career surgeons increasingly recognize not only what can be done, but what should be deferred, modified, or not attempted at all [6]. Their authority is quieter, less declarative, and therefore less visible within systems that reward assertiveness. Ironically, modern surgical systems may marginalize this wisdom [7]. Protocols, pathways, and performance metrics often leave little room for experiential judgment. Positioned between trainees and senior faculty, mid-career surgeons may recognize overconfidence in junior colleagues yet lack clearly sanctioned mechanisms to intervene without appearing obstructive or outdated. The erosion of longitudinal mentorship has further weakened the intergenerational transmission of epistemic humility [8].

Table 1 shows the evolution of confidence and judgment over different stages of a surgeon's career.

**Table 1:** Calibration of confidence and judgment across the surgical career

Career Stage	Dominant Source of Confidence	Common Cognitive Risk	Hallmarks of Surgical Judgment	Typical System-Level Reinforcement
Trainee/Early-career surgeon	Recent technical training, procedural fluency, and external validation	Overestimation of global competence; conflation of technical skill with judgment (Dunning–Kruger effect)	Focus on execution; reliance on rules and algorithms; limited appreciation of downstream consequences	Emphasis on autonomy milestones, case numbers, and decisiveness
Mid-career surgeon	Accumulated operative experience, independent outcomes, and professional recognition	Transitional miscalibration: confidence may exceed reflective self-awareness during peak productivity years	Contextual decision-making: growing attention to patient selection, timing, and alternatives to surgery	Rewards for volume, efficiency, leadership roles, and innovation
Late-career/Senior surgeon	Longitudinal experience, complication memory, and pattern recognition	Underestimation of expertise; perceived conservatism misread as hesitancy	Emphasis on restraint, proportionality of risk, and ethical judgment; knowing when not to operate	Reduced visibility of experiential wisdom within protocol-driven systems

Epistemic maturity and the ethics of surgical judgment

The Dunning-Kruger effect in surgery cannot be addressed solely at the level of individual psychology. It is structurally amplified by contemporary training and practice environments. Productivity metrics prioritize volume over deliberation. Innovation is valorized, while restraint remains invisible. Morbidity and mortality conferences, though essential, may drift toward ritualized defensiveness rather than genuine inquiry. Moreover, public and professional narratives increasingly equate excellence with fearlessness [9]. Conference podiums and digital platforms reward confidence signaling, reinforcing the illusion that certainty correlates with superiority. In such contexts, humility must be actively protected; it does not emerge spontaneously. The goal is not to suppress confidence, but to recalibrate it. Epistemic maturity in surgery involves aligning self-assessment with reality, recognizing uncertainty as intrinsic rather than shameful, and valuing restraint as a form of expertise. This requires cultural as well as structural change. Early-career surgeons must be supported in articulating doubt without reputational penalty. The more experienced must be empowered to mentor beyond technical instruction, modeling reflective judgment and intellectual honesty. Institutions must recognize that the safest surgeon is not the one who appears most certain, but the one whose confidence is proportionate to context and consequence.

Conclusions

The Dunning-Kruger effect in surgery is not an indictment of individual surgeons, but a reflection of the profession’s values. It reveals a persistent tension between decisiveness and doubt, autonomy and accountability, confidence and wisdom. Surgical practice requires confidence that is sufficiently strong to permit decisive action, yet sufficiently calibrated to accommodate uncertainty and evolving judgment. The most dangerous surgeon is not the novice who knows they are learning, but the competent surgeon who believes learning is complete. True mastery in surgery is marked not by the absence of doubt, but by the disciplined integration of it into every decision.
